# Diagnosing autism spectrum disorders using a double deep Q-Network framework based on social media footprints

**DOI:** 10.3389/fmed.2025.1646249

**Published:** 2025-08-20

**Authors:** Nesren S. Farhah, Ahmed Abdullah Alqarni, Nadhem Ebrahim, Sultan Ahmad

**Affiliations:** ^1^Department of Health Informatics, College of Health Science, Saudi Electronic University, Riyadh, Saudi Arabia; ^2^King Salman Center for Disability Research, Riyadh, Saudi Arabia; ^3^Department of Computer Sciences and Information Technology, Al-baha University, Al-baha, Saudi Arabia; ^4^Department of Computer Science, College of Engineering and Polymer Science, University of Akron, OH, United States; ^5^Department of Computer Science, College of Computer Engineering and Sciences, Prince Sattam Bin Abdulaziz University, Al-Kharj, Saudi Arabia; ^6^School of Computer Science and Engineering, Lovely Professional University, Phagwara, India

**Keywords:** autism spectrum disorders, diagnosing, social media, deep learning, disabilities, artificial intelligence

## Abstract

**Introduction:**

Social media is increasingly used in many contexts within the healthcare sector. The improved prevalence of Internet use via computers or mobile devices presents an opportunity for social media to serve as a tool for the rapid and direct distribution of essential health information. Autism spectrum disorders (ASD) are a comprehensive neurodevelopmental syndrome with enduring effects. Twitter has become a platform for the ASD community, offering substantial assistance to its members by disseminating information on their beliefs and perspectives via language and emotional expression. Adults with ASD have considerable social and emotional challenges, while also demonstrating abilities and interests in screen-based technologies.

**Methods:**

The novelty of this research lies in its use in the context of Twitter to analyze and identify ASD. This research used Twitter as the primary data source to examine the behavioral traits and immediate emotional expressions of persons with ASD. We applied Convolutional Neural Networks with Long Short-Term Memory (CNN-LSTM), LSTM, and Double Deep Q-network (DDQN-Inspired) using a standardized dataset including 172 tweets from the ASD class and 158 tweets from the non-ASD class. The dataset was processed to exclude lowercase text and special characters, followed by a tokenization approach to convert the text into integer word sequences. The encoding was used to transform the classes into binary labels. Following preprocessing, the proposed framework was implemented to identify ASD.

**Results:**

The findings of the DDQN-inspired model demonstrate a high precision of 87% compared to the proposed model. This finding demonstrates the potential of the proposed approach for identifying ASD based on social media content.

**Discussion:**

Ultimately, the proposed system was compared against the existing system that used the same dataset. The proposed approach is based on variations in the text of social media interactions, which can assist physicians and clinicians in performing symptom studies within digital footprint environments.

## 1 Introduction

**Figure 1:**
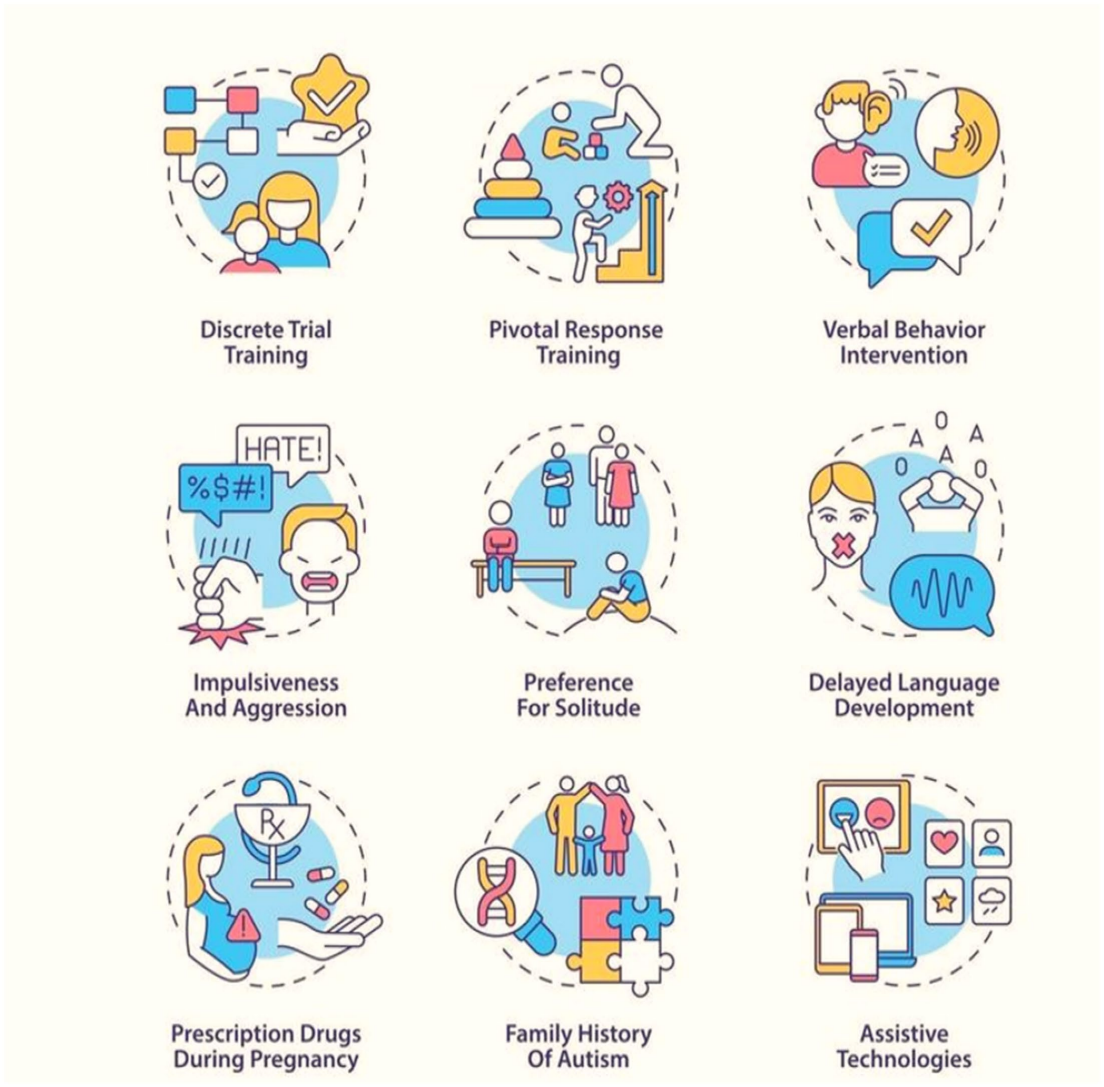
Displays the ASD concept.

ASD is among the most prevalent neurodevelopmental disorders. ASD is often demonstrated in children by age three and is defined by impairments in social interactions and communication, repetitive sensory-motor activities, and stereotypical behavioral patterns (1). ASD is a congenital neurodevelopmental condition characterized by symptoms that are evident in early infancy. Autism, characterized by restricted interests, repetitive behaviors, and significant disparities in social communication and interaction, typically emerges during early developmental stages and presents challenges in various social functioning domains. A child with autism induces significant anxiety within the family due to several factors, including the ambiguity of the diagnosis, the intensity and persistence of the disease, and the child’s nonconformity to social norms. In opposition, social awareness of autism is markedly inadequate, often conflated with intellectual disability and seen as an incurable ailment (2, 3). The ASD concept is displayed in Figure 1.

Content on social media, particularly videos and text disseminated by parents and caregivers, has emerged as a significant resource for facilitating the early identification of ASD (4, 5). Social media are technological tools designed for sharing, enabling users to create networks or engage in existing ones. In that order, the Pew Research Center identified the most popular social media sites as YouTube, Facebook, Instagram, Pinterest, LinkedIn, Snapchat, Twitter, and WhatsApp (6). Most consumers use these networks daily. This research utilizes Twitter data to assess the stigmatization of autism and associated terminology, picked based on accessibility and popularity, with analysis conducted using artificial intelligence technologies (7).

Conventional diagnostic methods, which primarily rely on observational and behavioral evaluations, often encounter issues with accessibility, consistency, and timeliness. Recent technology breakthroughs, especially in artificial intelligence (AI), and sensor-based techniques, provide novel opportunities for improving ASD identification. By developing more objective, accurate, and scalable approaches, these technologies transform diagnostic methodologies for autism spectrum disorder (ASD) (8–10). One new way to study the motor patterns, attentional processes, and physiological responses linked to ASD in real-time is wearable sensors, eye-tracking devices, and multimodal virtual reality settings. These technologies have the potential to give non-invasive, continuous monitoring, which might help with the early diagnosis of ASD and shed light on neurological and behavioral traits that have been hard to document reliably.

Nevertheless, advancements in contemporary research are required to substantiate their efficacy. Sensor-based techniques may facilitate the identification of stereotyped behaviors and motor patterns linked to ASD in realistic environments, potentially yielding data that could guide timely and customized therapies (11). Neuroimaging and microbiome analysis further advance this technical domain by indicating neurological and biological traits specific to ASD. AI-enhanced neuroimaging aids in identifying structural and functional brain connection patterns associated with ASD, thereby enhancing the understanding of its neuroanatomical foundation (12).

The research conducted by Neeharika and Riyazuddin et al. (13) aimed to enhance the accuracy of ASD screening by using feature selection methods in conjunction with sophisticated machine learning classifiers. Their research included several datasets spanning infants, children, adolescents, and adults, enabling a thorough assessment of ASD characteristics across different age demographics. Authors’ use of MLP model capacity to reliably and rapidly identify ASD, indicating a beneficial screening instrument suitable for various age groups, facilitating both clinical evaluations and extensive screenings. Wall et al. (14) investigated machine learning (ML) algorithms for diagnosing ASD using a standard dataset. The researchers focused on the Alternating Decision Tree classifier to identify a limited yet efficient set of queries that optimize the diagnostic procedure. Alzakari et al. (15) proposed a novel two-phase methodology to tackle the variability in ASD features with ML approaches, including behavioral, linguistic, and physical data. The first step concentrates on identifying ASD, using feature engineering methodologies and ML algorithms, including a logistic regression (LR) and support vector machine (SVM) ensemble, attaining a classification with high accuracy. EEG assesses brain activity and may identify children predisposed to developing ASD, hence facilitating early diagnosis. EEG data is used to compare ASD and HC (16–18). In (19), the CNN model was used for classification after transforming the data into a two-dimensional format. While EEG may facilitate the diagnosis of ASD, it is constrained by other factors, such as signal noise.

The research has used social media to investigate ASD. However, exploiting these prevalent platforms and innovative online data sources may be feasible to enhance the comprehension of these diseases. Previous research has utilized Twitter data to investigate discussions on ASD-related material, indicating that this subject is frequently addressed on this platform (20). Considering the use of social media for researching ASD is particularly significant, as a recent analysis indicated that around 80% of individuals with ASD engage with prominent social media platforms (21). This study aims to build upon previous research and enhance our comprehension of whether publicly accessible social media data from Twitter may provide insights into the existence of digital diagnostic indicators for ASD (22). Furthermore, we want to assess the viability of establishing a digital phenotype for ASD using social media.

Beykikhoshk et al. (20) examined Twitter’s potential as a data-mining tool to comprehend the actions, challenges, and requirements of autistic individuals. The first finding pertained to the attributes of participants inside the autism subgroup of tweets, indicating that these tweets were highly informative and had considerable potential usefulness for public health experts and policymakers. Tomeny et al. (23) examined demographic correlations of autism-related anti-vaccine opinions on Twitter from 2009 to 2015. Their results indicated that the frequency of autism-related anti-vaccine views online was alarming, with anti-vaccine tweets connecting with news events and demonstrating geographical clustering. From 2015 to 2019, Tárraga-Mínguez et al. (24) examined the phrases “autism” and “Asperger” in Spain in relation to Google search peaks. The public view of autism was significantly impacted by how the condition was portrayed in the news and on social media, and the authors found that social marketing campaigns had a significant role in normalizing autism. In this research (25), looked at how people sought assistance. The results showed a strong correlation in Google search interest between the terms “Asperger syndrome” and “Greta Thunberg,” reaching their highest point in 2019. Online traffic to the Asperger/Autism Network and Autism Speaks websites increased steadily from June to December 2019, indicating a correlation between help-seeking behavior and Thunberg’s fame, according to the research. According to the results, the stigma associated with Asperger’s disorder may have been positively affected by Thunberg’s public exposure.

### 1.1 Contribution

The use of tweets from Twitter for the detection of ASD is substantial, since it offers extensive, real-time, user-generated data that facilitates the early identification of ASD-related behaviors, particularly via self-reported experiences and parental observations. This methodology promotes the advancement of suggested models, namely LSTM, CNN-LSTM, and inspired DDQN, for natural language processing to examine linguistic patterns, feelings, and keywords related to ASD. It provides insights into popular views, stigma, and misconceptions around autism, guiding awareness initiatives and public health measures. Twitter data is a powerful and accessible resource for enhancing early detection and understanding of ASD in diverse groups. Utilizing social media in this manner may offer more accessible and timely screening, particularly in regions with limited healthcare resources.

## 2 Materials and methods

**Figure 2:**
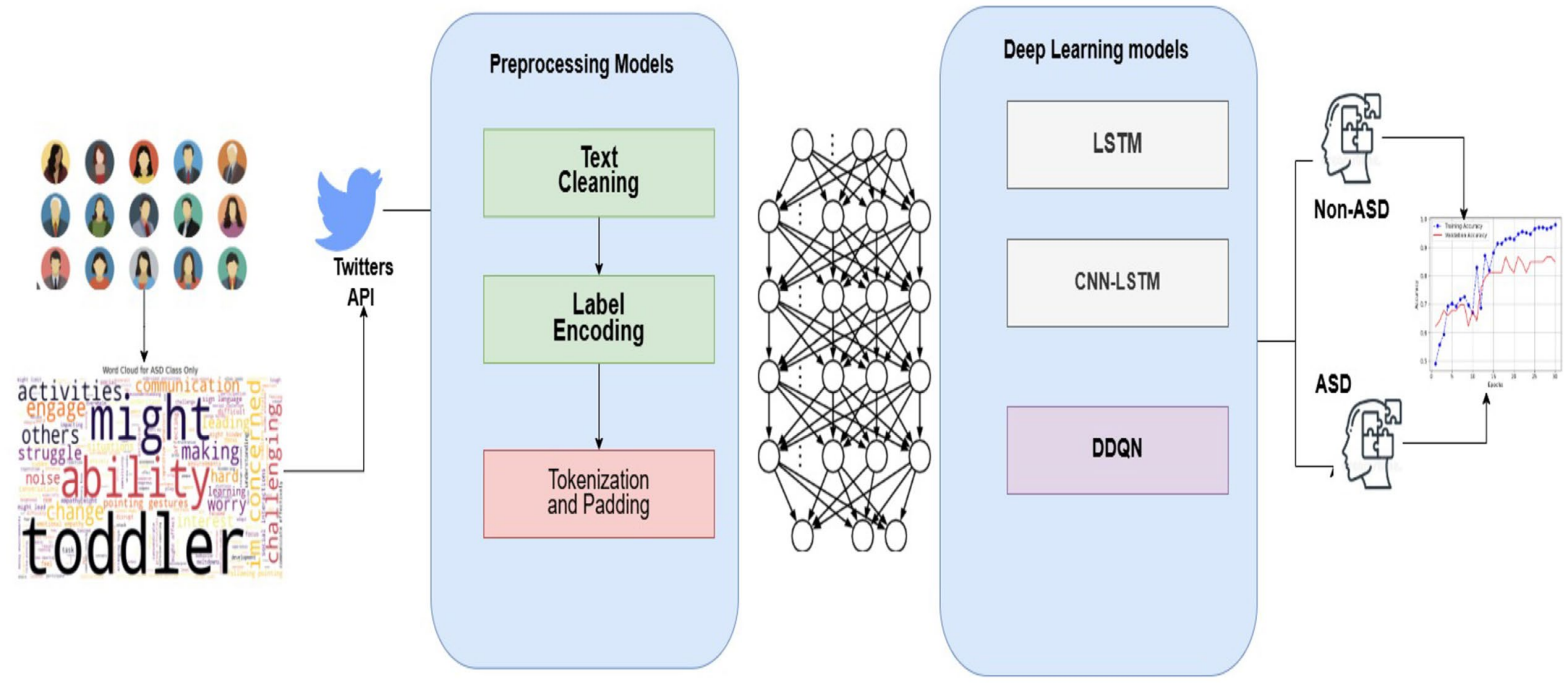
Farmwork of ASD system.

Figure 2 shows the pipeline of the proposed system to provide a broader perspective to researchers and developers. The framework delineates the processing phases for the pipeline that utilizes social media content to diagnose ASD. Below, we present a comprehensive assessment of each step.

### 2.1 Dataset

**Figure 3:**
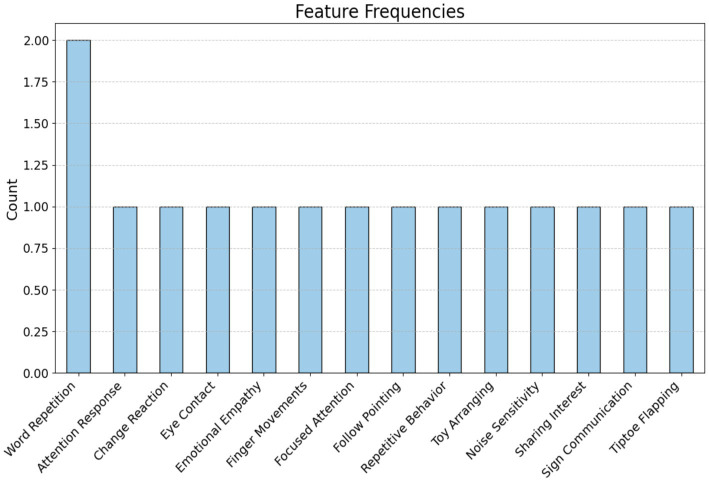
Features of the dataset.

**Figure 4:**
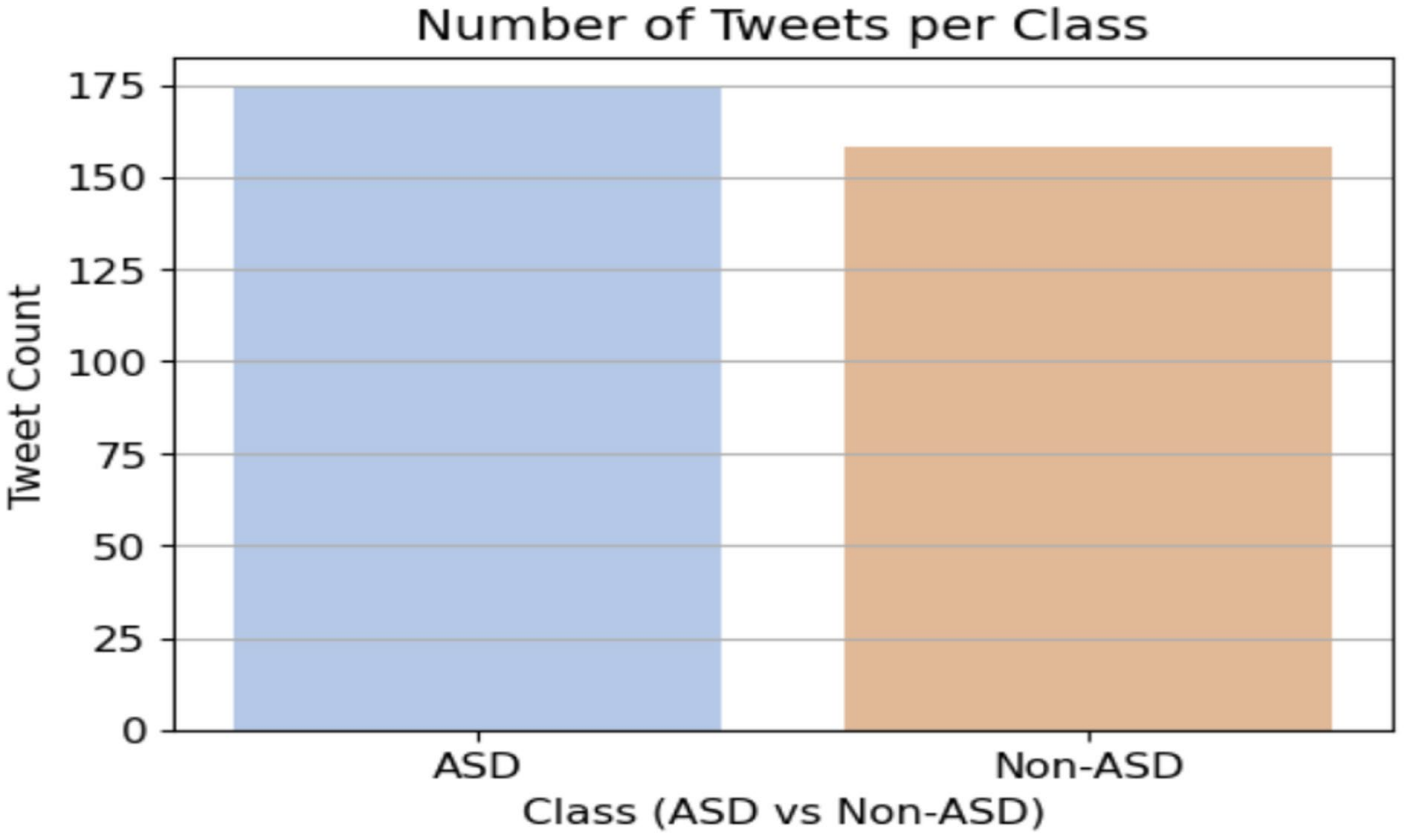
Label of the dataset.

To help with the early diagnosis of ASD by using proposed systems, the TASD-Dataset includes comprehensive textual sequences that depict the everyday lives of children with and without ASD. It offers new elements, including Noise Sensitivity, Sharing Interest, Sign Communication, and Tiptoe Flapping, It combines critical ASD assessment aspects like Attention Response, Word Repetition, and Emotional Empathy, as shown in Figure 3. Parents may get detailed insights and better identify signs of autism spectrum disorder (ASD) due to the deepening of certain behaviors. The dataset contains 172 tweets from the ASD class and 158 non-ASD tweets. Figure 4 shows the class of the dataset.

### 2.2 Preprocessing

**Figure 5:**
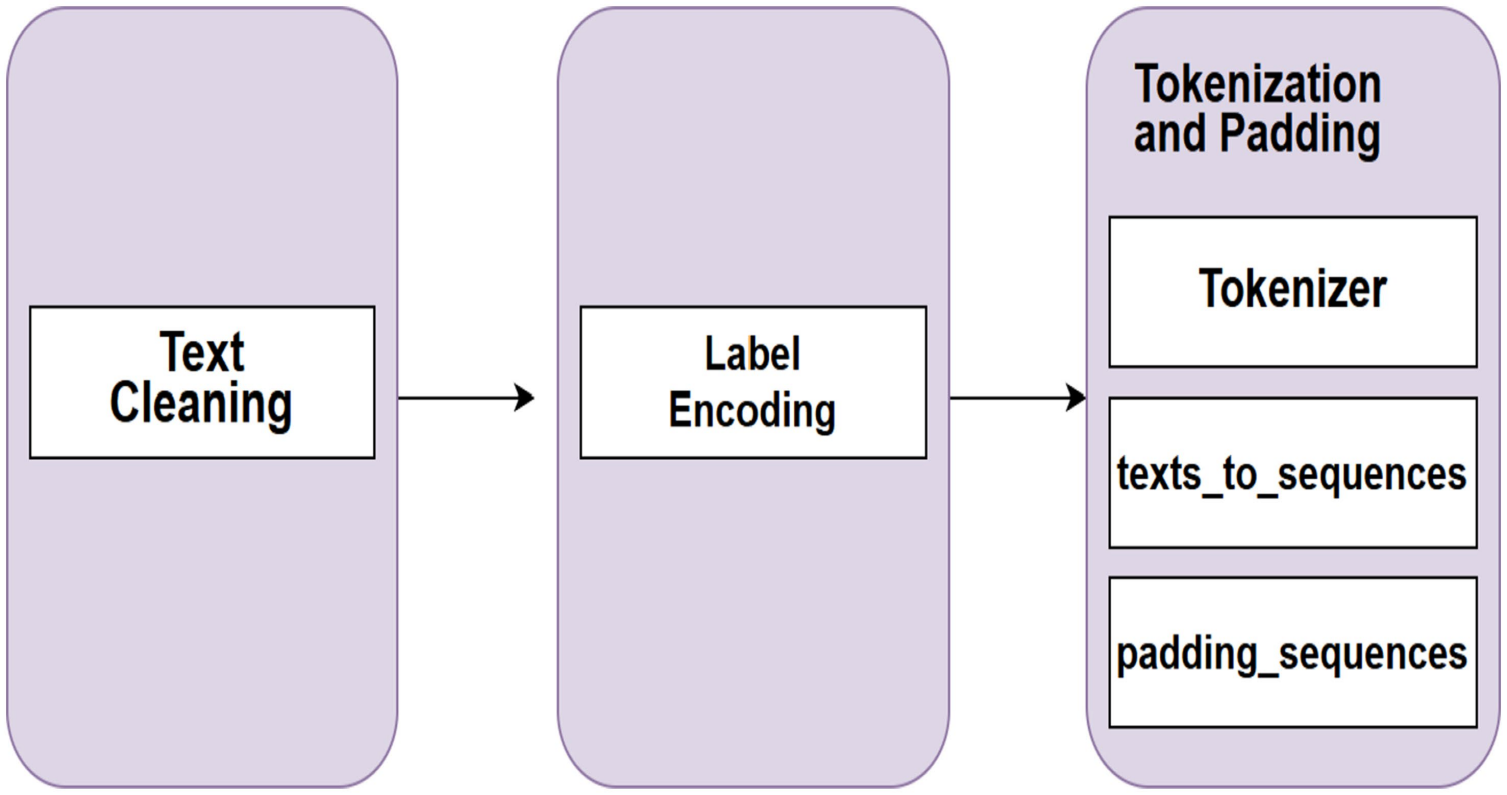
Preprocessing ASD text analysis.

Text preprocessing is an essential step in the text processing process. Words, sentences, and paragraphs can all be found in a text, which is defined as a meaningful sequence of characters. Preprocessing methods feed text data to a proposed algorithm in a better form than in its natural state. A tweet can contain different viewpoints on the data it represents. Tweets that have not been preprocessed are highly unstructured and contain redundant data. To address these issues, several steps are taken to preprocess tweets for detecting ASD, as shown in Figure 5.

### 2.3 Text cleaning

**Figure 6:**
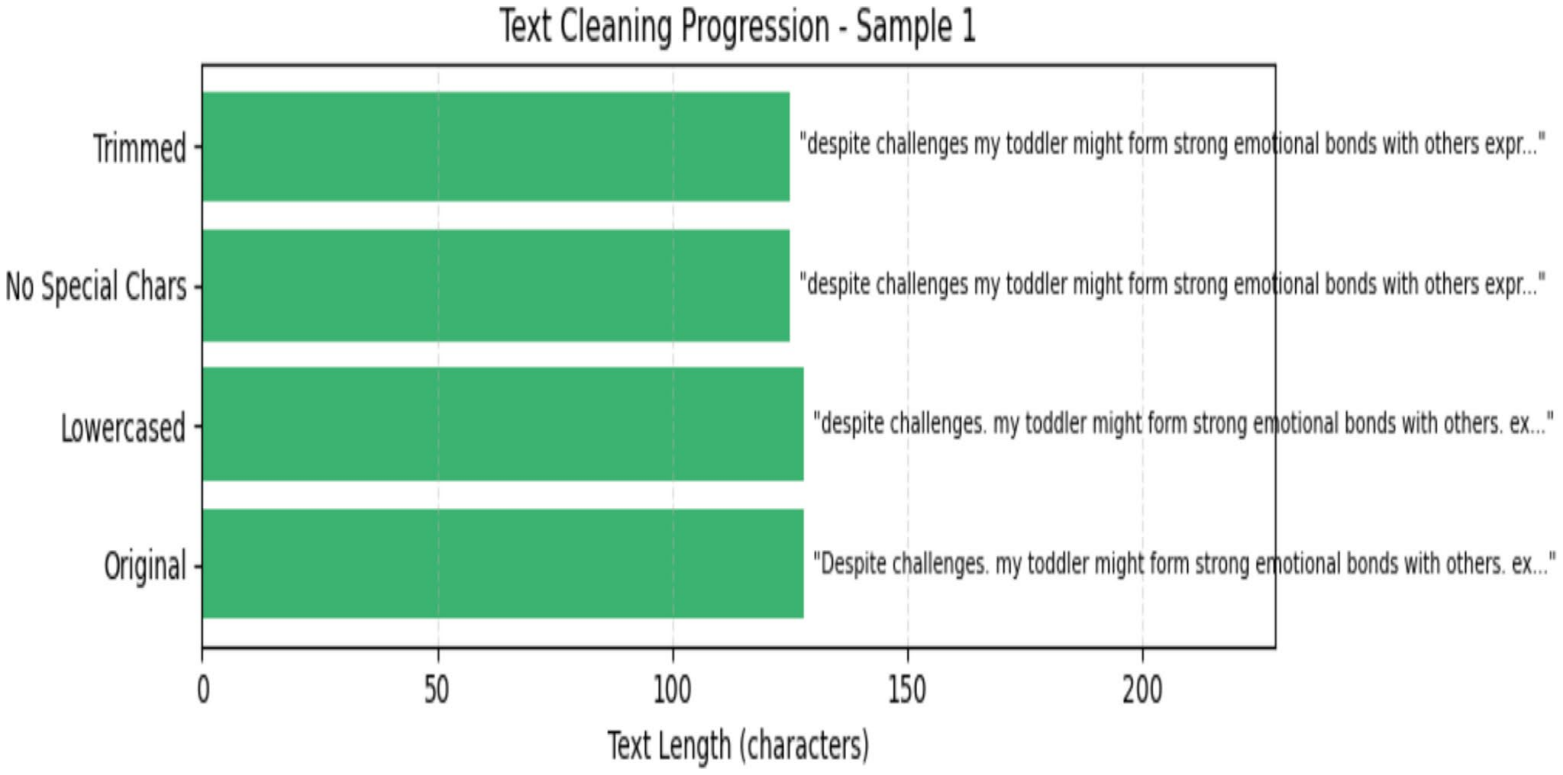
Clean text.

The clean text preprocessing method is a significant step in text datasets because the text contains several extra contexts to preprocess and normalize raw text data for analysis. In these steps, the use is transformed to lowercase to guarantee consistency and prevent differentiation between “ASD” and “Non-ASD.” Subsequently, any characters that are not letters, numerals, or spaces are eliminated by a regular expression, so punctuation and other symbols that might create extraneous noise are removed. This method is ultimately applied to the ‘Text’ column of the Data Frame, ensuring that all text elements are sanitized and prepared for feature extraction. Figure 6 displays the clean text process.

### 2.4 Label encoding

The LabelEncoder method converts text class (ASD and Non-ASD) into numbers, designating 0 for ASD and 1 for Non-ASD. This transformation updates the classification effort by enabling the model to see the labels as numerical values instead of text. Equations 1, 2 show the label encoding.


(1) yclassification∈(ASD,Non−ASD)Then

(2) y=labelEncoder(yclassifcation)→y∈{0,1}

### 2.5 Tokenization and padding

**Figure 7:**
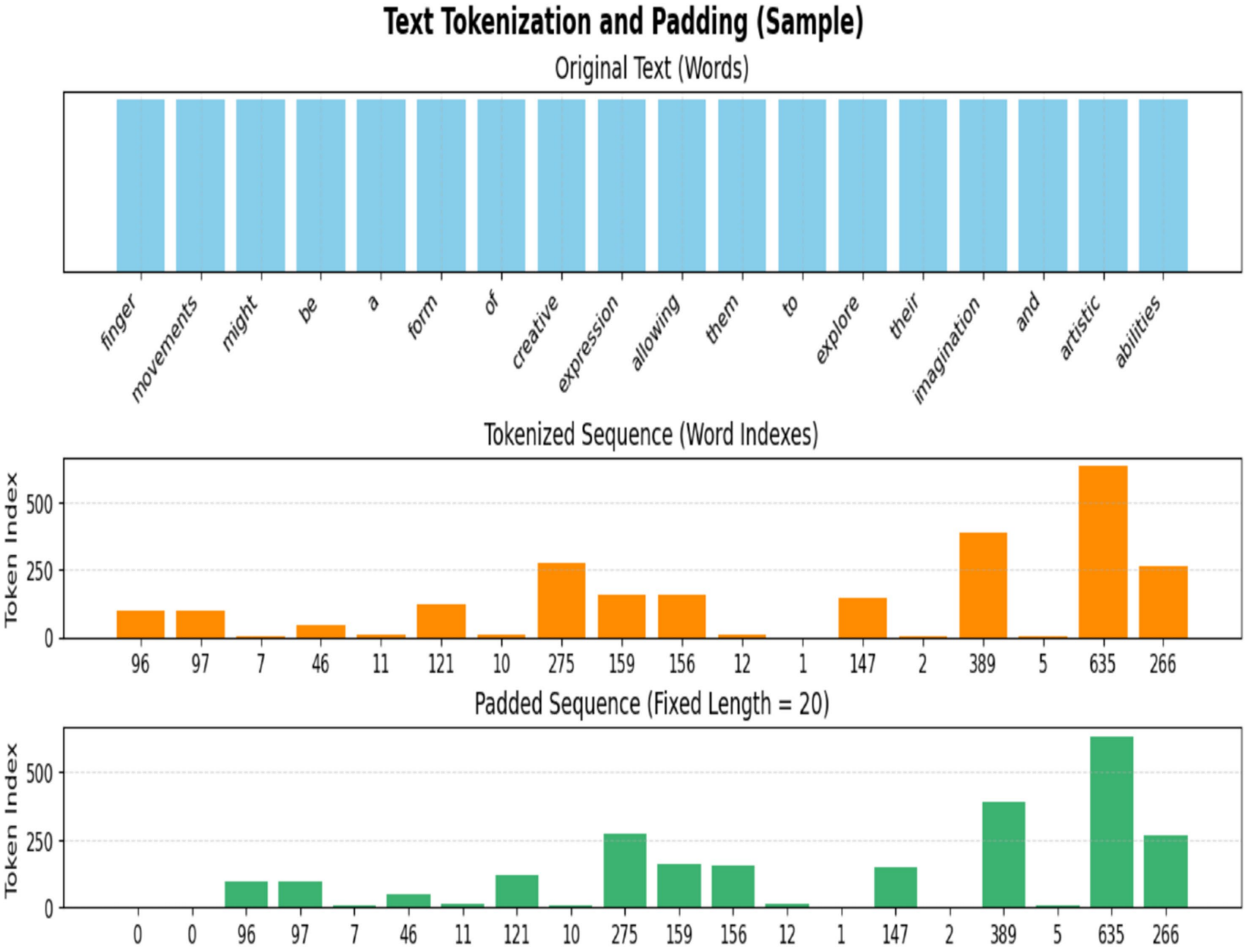
Sample of text tokenization and padding.

Tokenization and padding are essential NLP preprocessing procedures that transform unprocessed text into a numerical representation appropriate for machine learning models, particularly neural networks. Figure 7 shows the tokenization and padding Equation 3.

#### 2.5.1 Tokenizer

**Figure 8:**
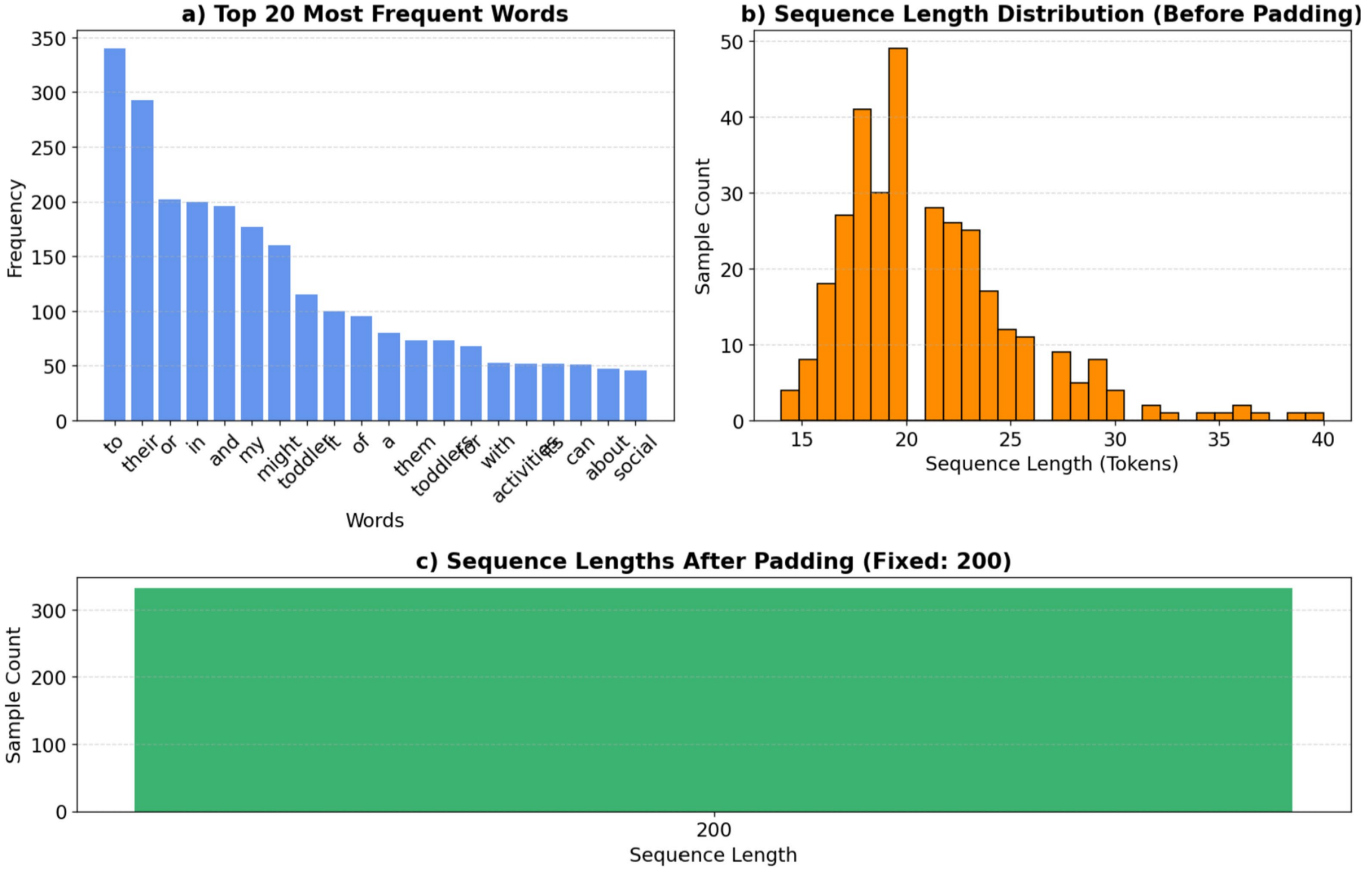
Tokenizer analysis: word frequencies and sequence lengths.

Tokenizer procedures transform textual data into a numerical representation suitable for input into neural networks. They convert a text corpus into integer sequences, assigning a distinct index to each unique word according to its frequency, as shown in Equation 3. The tokenizer processing is shown in Figure 8.


(3) $$\text{index}(w)={\mathrm{rank}}_{f}(w)\;\text{if}\;{\mathrm{rank}}_{f}(w)\leq V$$

Where ${\mathrm{rank}}_{f}(w)\;$is rank w frequency f(w) and V is the maximum number of words.

#### 2.5.2 Fit texts

This phase is crucial for transforming unprocessed text into numerical sequences suitable for input into the proposed system.

#### 2.5.3 Texts_to_sequences

To convert unprocessed text input into sequences of word indices according to the mapping acquired via as shown in Equation 4.


(4) $$\text{sequence}(T_{i})=[\text{index}(w_{1}),\text{index}\;(w_{2}),\ldots \ldots \ldots ,\text{index}\;(w_{m})]\;$$

Where is the $T_{i}$ is the sentence of the text contained, and w is the words of the text, whereas the $\text{index}(w_{1})$ is an index of the words in the context.

#### 2.5.4 Padding_sequences

Normalize sequence lengths, which may differ post-tokenization, by padding shorter sequences and truncating larger ones to a predetermined length as shown in Equation 5. The padding and truncated b are fixed on the length. L=200. The padding processing is shown in Figure 7.


(5) $$x=|\begin{array}{c} x_{1}\\ x_{2}\\ \begin{array}{c} .\\ .\\ .\\ . \end{array}\\ y \end{array}|\in {\mathbb{R}}^{\mathrm{nxL}}$$

Where x is features contain padding and are tokenized, L is the length of the vector. The number of texts is indicated n, and $\in {\mathbb{R}}^{\mathrm{nxL}}$ is matrix lues.

### 2.6 Proposed systems

#### 2.6.1 Convolutional neural networks

**Figure 9:**
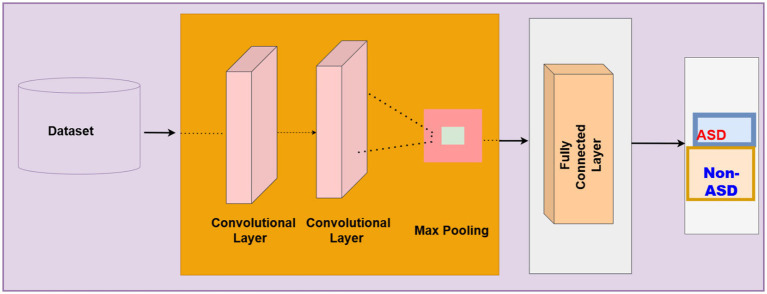
Structure CNN.

The CNN model is at the core of all advanced machine learning and deep learning applications. They can successfully address text classification, image recognition, object identification, and semantic segmentation. Using the same method with a task as different as Natural Language Processing is counterintuitive (7). The structure is presented in Figure 9. Equation 6 presents the convolution layer of CNN.


(6) $$O(x,y)=\sum_{i=1}^{H}\;\sum_{j=1}^{W}\;I(x+i,y+j)*K(i,j)+b$$

Where the features of text O(x,y) The feature of the text is mapped by using.I(x+i,y+j) is weighted by a neural network and b is biased to adjust the neural. The ReLU activation function is Equation 7, the max pooling function is presented in Equation 8. The Dense Layer is given in Equation 9.


(7) f(x)=max(0,x)

(8) $$O(x,y)=\sum_{i=1}^{H}\;\sum_{j=1}^{W}\;I(x+i,y+j)*K(i,j)+b$$

(9) O=W·X+b

#### 2.6.2 Long short-term memory network

**Figure 10:**
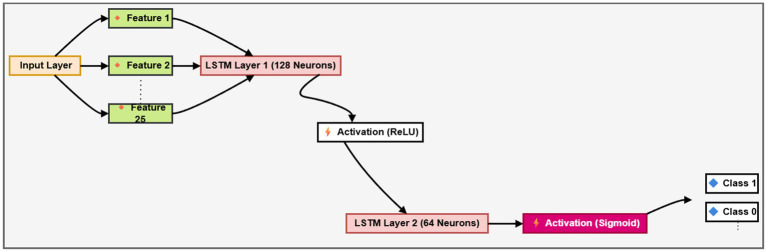
LSTM model.

An LSTM network is an advanced form of a sequential neural network. It fixes the problem of RNN gradients fading over time. RNNs often handle long-term storage. At a high level, the operation of an LSTM is comparable to that of a single RNN neuron. The inner workings of the LSTM network are outlined in this section. The LSTM consists of three parts, each performing a particular function, as seen in Figure 10 below. In the first step, it is decided whether the information from the previous time stamp is significant enough to be saved or if it is harmless enough to be deleted. In the second step, the cell will try to acquire new information by analyzing the data that has been presented to it. In the third and final step, the cell incorporates the data from the most recent time stamp into the data stored in the next time stamp. These three components constitute what is referred to as a gate for an LSTM cell. The “Forget” gate comes first, followed by the “Input” section, and then the “Output” section is used to define the last portion as shown in Equations 10– 14.


(10) $$\text{Forget gate}:f_{t}=\sigma (W_{f}.X_{t}+W_{f}.h_{t-1}+b_{f})$$

(11) $$\text{Input gate}:i_{t}=\sigma (W_{c}.X_{t}+W_{i}.h_{t-1}+b_{i})$$

(12) $$\text{Cell gate}:C_{t}=(W_{f}*(.h_{t-1},x_{t})\;b_{f})$$

(13) $$\text{Output gate}:o_{t}=\sigma (W_{o}+X_{t}+W_{o}.h_{t-1}+V_{o}.C_{t}+b_{o})$$

(14) $$\text{Hidden layer}:h_{t}=o_{t}+\tanh(C_{t})$$

In Figure 10, $C_{t}$ Represent the prior and current states of the cell, respectively. Both $\;h_{t-1}$ and h Represents the cell output that was processed before the one now being processed. It is common practice to disregard $f_{t}$ As a gate, even though it is the input gate. The output of a sigmoid gate is symbolized here by $o_{t}$. The cable that connects the cell gates is where all the data collected by the cell gates is sent to and from C. The $f_{t}$ Layer decides to remember anything, and the$\;f_{t}\text{The}\;$Output is multiplied by c to do so (t-1). After that, c (t-1) is multiplied by the product of the sigmoid layer gate and the tanh layer gate, and the output h t is generated by point-wise multiplication of $o_{t}$ and tanh.

The LSTM architecture is intended to capture long-term relationships in Twitter text data. The preprocessing converts input words that start with an embedding layer into 128-dimensional dense vectors. The LSTM layer with 64 units is then used to mitigate overfitting, integrating dropout and recurrent dropout with 0.5. An L2 regularization term is further included in the LSTM and output dense layer. Table 1 shows parameters of the LSTM model.

**Table 1:** LSTM parameters model.

Input	Values
Embedding dimension	256
LSTM unit	64
Conv1D	64, K = 5
MaxPooling ID	yes
Dropout_rate	0.5
Dense_Unites	32
Activation_function	ReLU
L2	0.001
Optimizer	Adam
Loss	Binary
Epoch	30
Batch size	16

#### 2.6.3 CNN-LSTM model

The CNN-LSTM model is a hybrid architecture that combines convolutional neural networks (CNN) for spatial feature extraction and long short-term memory (LSTM) networks for sequential learning, making it highly effective for analyzing text data such as tweets. The model begins with an embedding layer that transforms each word into a 256-dimensional dense vector, capturing the semantic meaning of words. This is followed by a 1D convolutional layer with 64 filters and a kernel size of 5, which scans through the text to detect local patterns and n-gram features such as common word combinations or phrases often associated with ASD. A batch normalization layer is applied to stabilize and accelerate training, followed by a max pooling layer that reduces the dimensionality and computational load by selecting the most prominent features. A dropout layer with a rate of 0.5 is then used to prevent overfitting by randomly deactivating some neurons during training. The output is passed into a 64-unit LSTM layer that captures the temporal dependencies and contextual relationships across the tweet sequence. Finally, a dense layer with sigmoid activation performs binary classification to predict whether the tweet indicates ASD-related content. The model is trained using the Adam optimizer, binary cross-entropy loss, class weights, and regularization to handle imbalanced data and improve generalization. The critical parameters of the CNN-LSTM model are displayed in Table 2.

**Table 2:** CNN-LSTM parameters.

Input	Values
Embedding dimension	128
LSTM unit	64
Conv1D	No
MaxPooling ID	No
Dropout_rate	0.5
Dense_Unites	32
Activation_function	ReLU
L2	0.001
Optimizer	Adam
Loss	Binary
Epoch	30
Batch size	16

#### 2.6.4 Double deep Q-network (DDQN-inspired)

The Double Q-Learning model was introduced by H. van Hasselt in 2010, addressing the issue of significant overestimations of action value (Q-value) inherent in traditional Q-Learning. In fundamental Q-learning, the Agent’s optimal strategy is consistently to select the most advantageous action in any specific state. This concept’s premise is that the optimal action corresponds to the highest expected or estimated Q-value. Initially, the Agent lacks any knowledge of the environment; it must first estimate Q(s, a) and subsequently update these estimates with each iteration. The Q-values exhibit considerable noise, leading to uncertainty about whether the action associated with the highest expected or estimated Q-value is genuinely the optimal choice.

**Figure 11:**
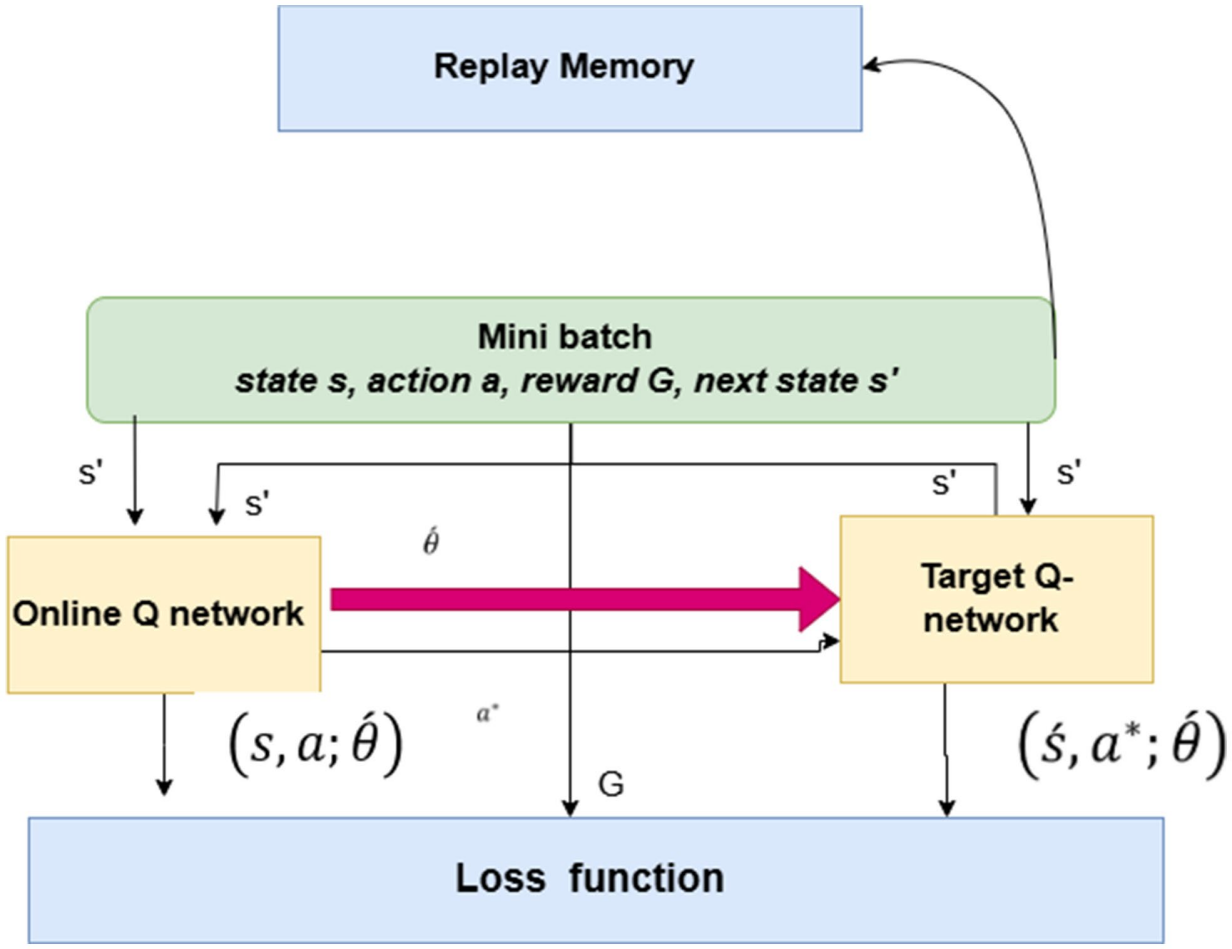
DDQN-inspired model.

Double Q-Learning employs two distinct action-value functions, Q and Q’, as estimators. Even if Q and Q’ exhibit noise, this noise can be interpreted as a uniform distribution as shown Figure 11 The update procedure exhibits some variations compared to the basic version. The action selection and action evaluation processes are separated into two distinct maximum function estimators. shown in Equations 15, 16.

Let the vector of a neural network’s weights be represented by *θ*. We establish two Q-networks: the online Q-network Q (s, a; θ(t)) and the target Q-network Q (s, a; θ (t)). To be more specific, the training of Q (s, a; *Χ* (t)) is done by modifying the weights (t) at time slot t in relation to the goal value y(t).


(15) $$y(t)=G(t)+(s\prime ,\text{argmax}Q(s\prime ,a^{*};\theta \prime (t));\theta \prime (t))$$

(16) $$y(t)=G(t)+(s\prime ,\text{argmax}Q(s\prime ,a^{*};\theta \prime );\theta_{i-1})$$

The reinforcement learning mechanism integrates generative artificial intelligence for decision-making and prediction tasks, as shown in Equations 15, 16. This equation indicates the generative which produces the estimation or hypothesis at a given time t.DoubleQ−LearningUsed next state, whereas the s’ is exit state and $\text{argmax}Q\;(s\prime ,a^{*};\theta \prime (t))$ defined as the action of $a^{*}$ to maximize the predicted Q-value based on the current parameters. To estimate the Q-value of this selected action in the next state, the outer Q-function Q’ employs the older parameters. $\theta_{i-1}$1, which helps reduce overestimation bias. This combination makes applications for predicting ASD from social media content domains possible.

The DDQN model is used to classify ASD and non-ASD cases utilizing text data. The model utilizes a preprocessing step for text processing that encompasses data loading, cleaning (including lowercasing, removal of special characters, and normalization of spaces), and tokenization, constrained by a maximum vocabulary of 10,000 words and a sequence length of 200. The model architecture, drawing from the Double Deep Q-Network (DDQN) model comprises an input layer, an embedding layer with 256 dimensions, and two parallel LSTM branches, each containing 64 units, a dropout rate of 0.5, and L2 regularization to capture sequential patterns effectively. The model uses the Adam optimizer with a learning rate of 1e-4 and employs binary cross-entropy loss. It is trained for 30 epochs, incorporating early stopping and learning rate reduction callbacks to mitigate overfitting. Parameters of DDQN-Inspired are shown in Table 3.

**Table 3:** Parameters of DDQN-inspired.

Input	Values
Max-sequence length	200
Vocabulary	10,000
Embedding_dimension	256
Dropout_rate	0.5
Dense_Unites	32
Activation_function	ReLU
LS	0.0001
Optimizer	Adam
Loss	Binary
Epoch	30
Batch size	16

## 3 Performance of the framework

### 3.1 Performance of LSTM

**Figure 12:**
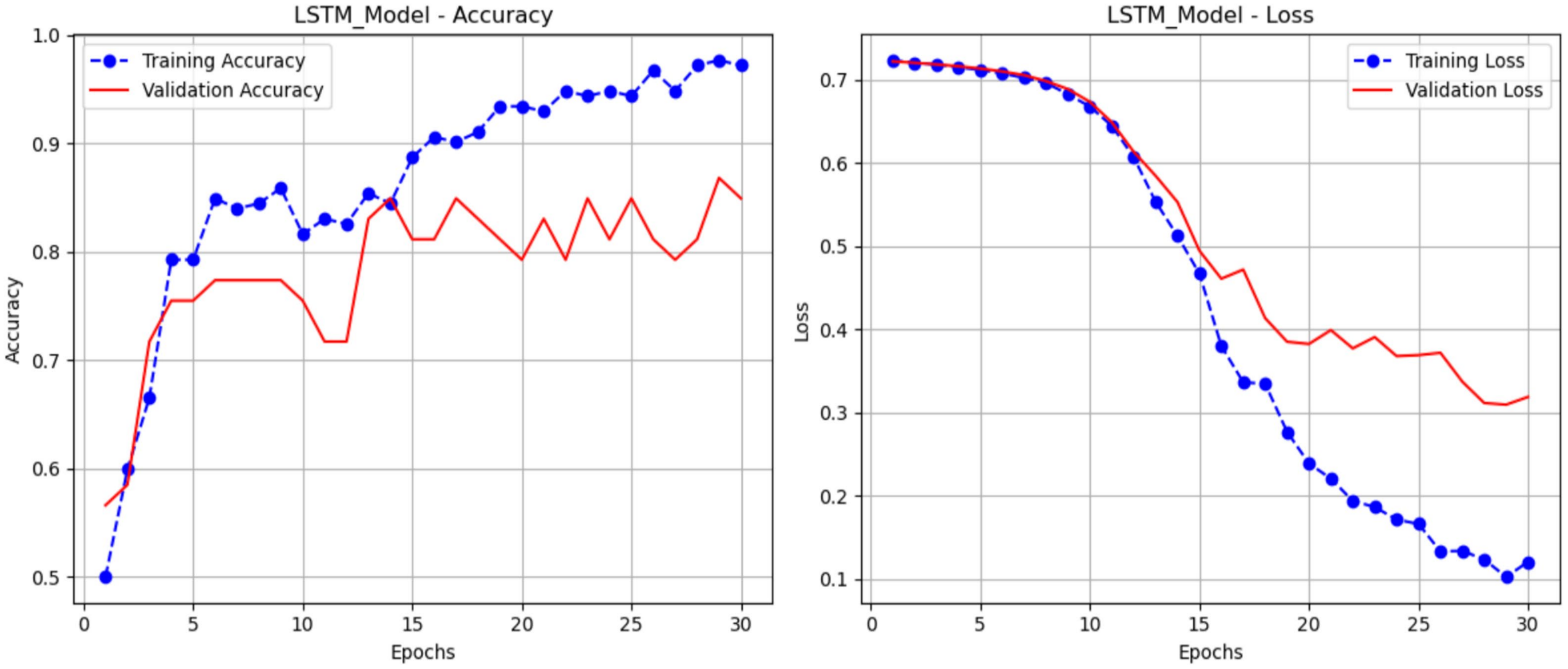
Performance of the LSTM model.

Figure 12 presents the accuracy and loss metrics used to train and validate an LSTM model over 30 epochs. The validation accuracy of the LSTM model, displayed in red, begins at a lower value and increases to about 81%. The blue line in the accuracy plot (a) shows the training accuracy of the LSTM model; it increases gradually from around 50% to almost 99%, showing that the model learns the training data well over time. The plot (b) shows the loss of the LSTM model; the blue line represents the training loss, which drops gradually from around 0.7 to less than 0.2, suggesting that the model is getting a better fit to the training data. Meanwhile, the red validation loss line declines from around 0.7 to about 0.3. While the training loss continues to grow, the validation loss reaches a level and exhibits small oscillations, suggesting that the model’s generalizability may stabilize.

**Figure 13:**
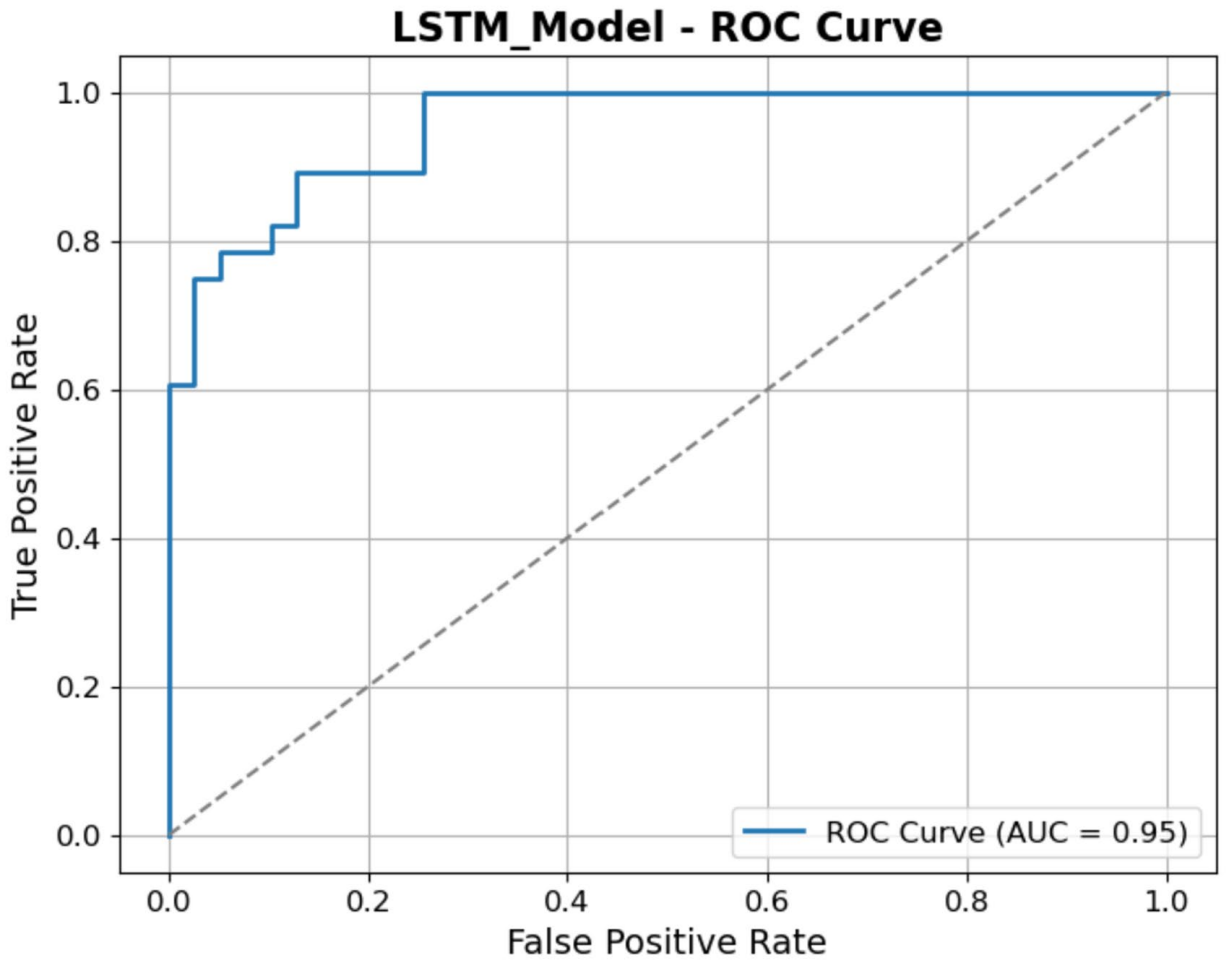
ROC of the LSTM model.

The ROC curve illustrated in Figure 13 shows the efficacy of the LSTM model in differentiating between the classes. The graph illustrates the TP rate (sensitivity) in relation to the FP Rate across different threshold levels. The LSTM model attains an AUC of 0.95, demonstrating exceptional classification capability. The AUC of 1.0, but a result of 0.5 indicates random chance.

### 3.2 Performance of the CNN-LSTM model

**Figure 14:**
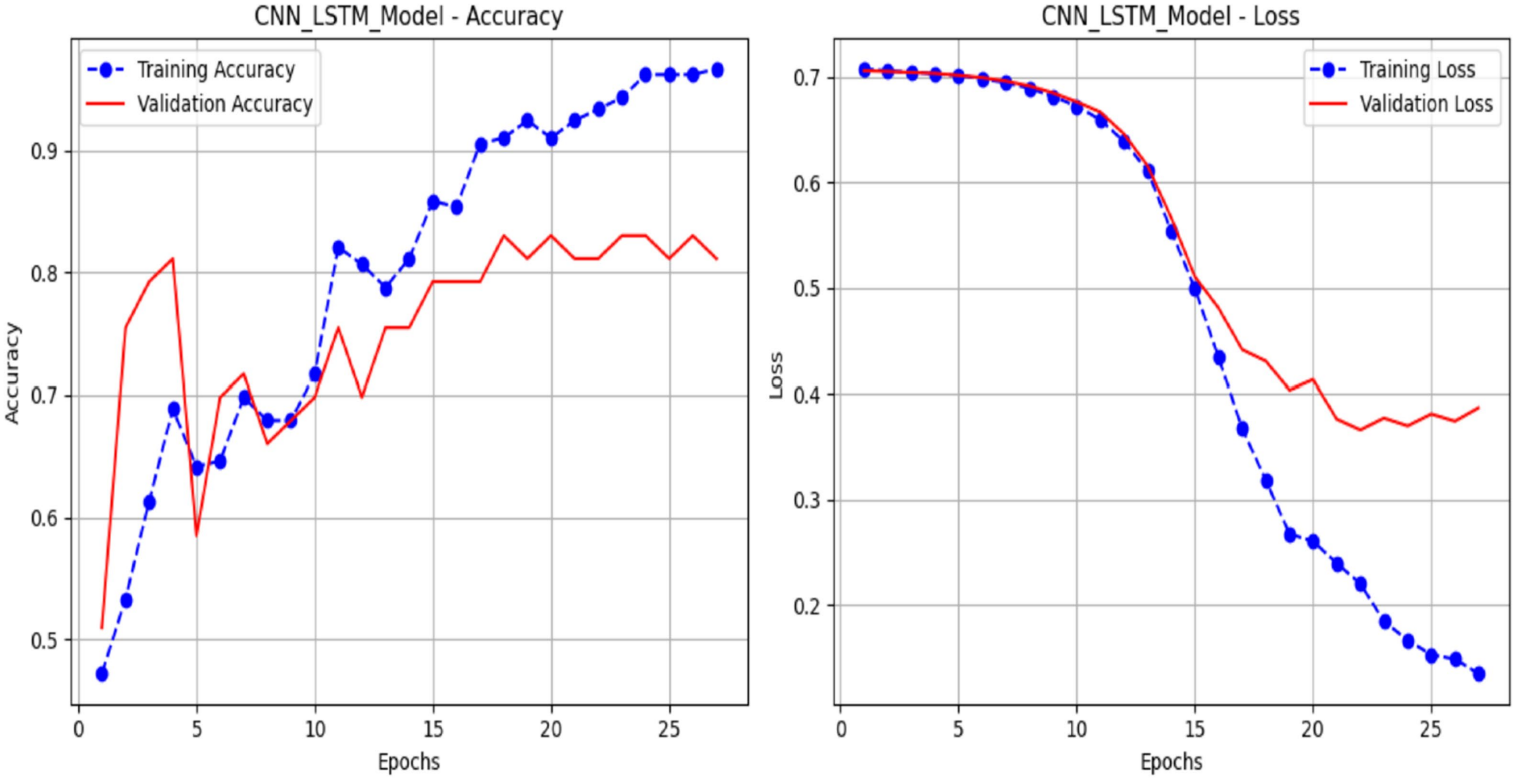
Performance of the LSTM model.

Figure 14 presents plots illustrating the performance of a CNN-LSTM model over 25 epochs, showing its training and validation metrics for accuracy and loss. The accuracy plot (a) illustrates the training accuracy (blue line), which increases progressively from approximately 51.42% to nearly 99.53%, indicating effective learning from the training data. In contrast, the validation accuracy (red line) rises to about 83.02% with some variability, indicating satisfactory but imperfect generalization. The loss plot (b) shows the training loss (blue line) declining steadily from 0.7140 to below 0.0760, indicating enhanced model fit. In contrast, the validation loss (red line) decreases from 0.7130 to approximately 0.3530, with a slight decline toward the conclusion. This notification indicates that the CNN-LSTM model demonstrates efficient learning, as evidenced by the difference between the training and validation measures.

**Figure 15:**
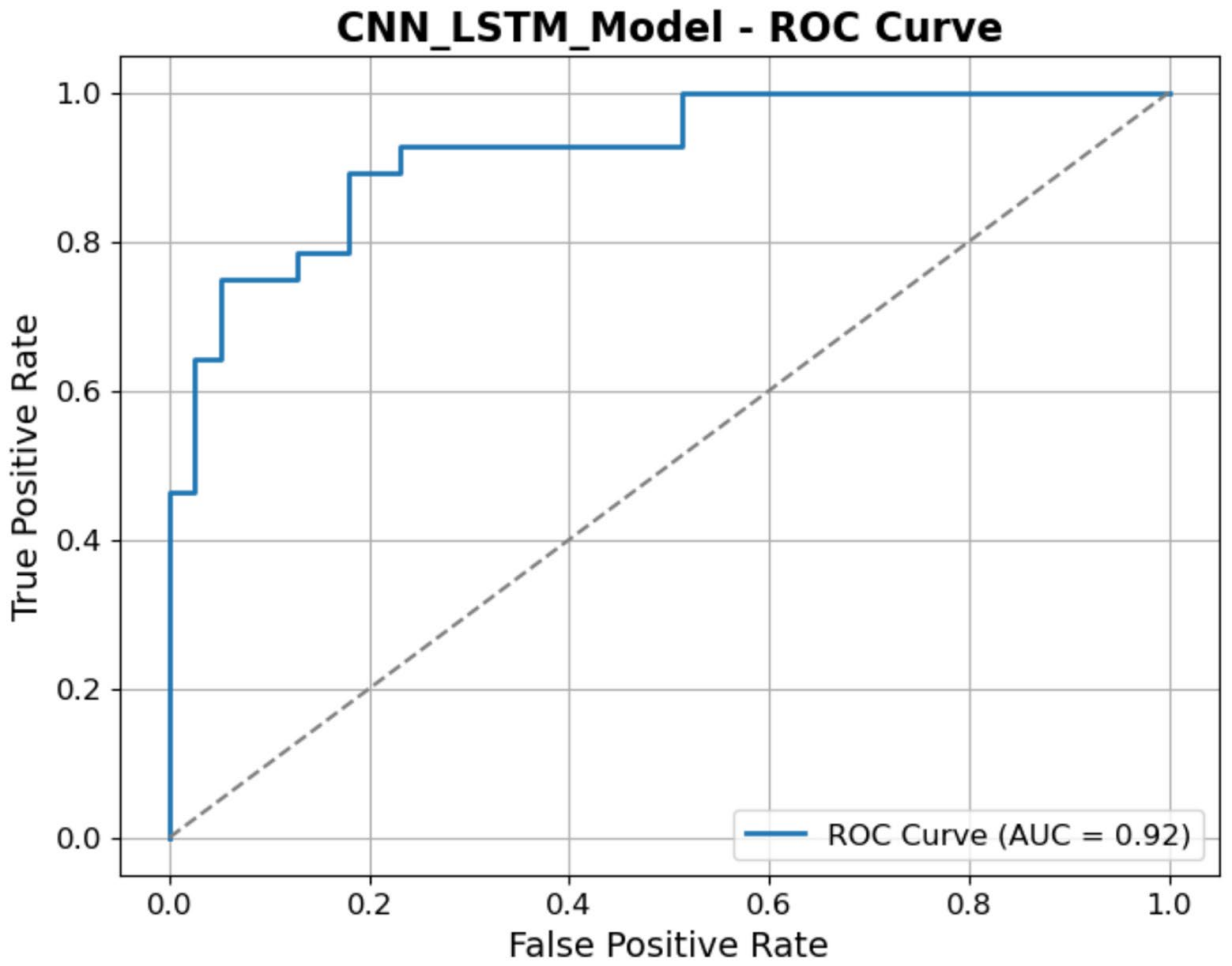
ROC of the CNN-LSTM model.

Figure 15 illustrates the ROC curve for the CNN-LSTM model, illustrating its classification performance at various thresholds. The graph illustrates the TP Rate (Sensitivity) in relation to the FP Rate, with the AUC recorded at 92%. The elevated AUC value indicates the model has robust discriminative capability in differentiating between the ASD and Non-ASD classes. The ROC ascends rapidly toward the top-left corner, as seen in the figure, indicating a high TP rate with few false positives.

### 3.3 Performance of DDQN-inspired model

**Figure 16:**
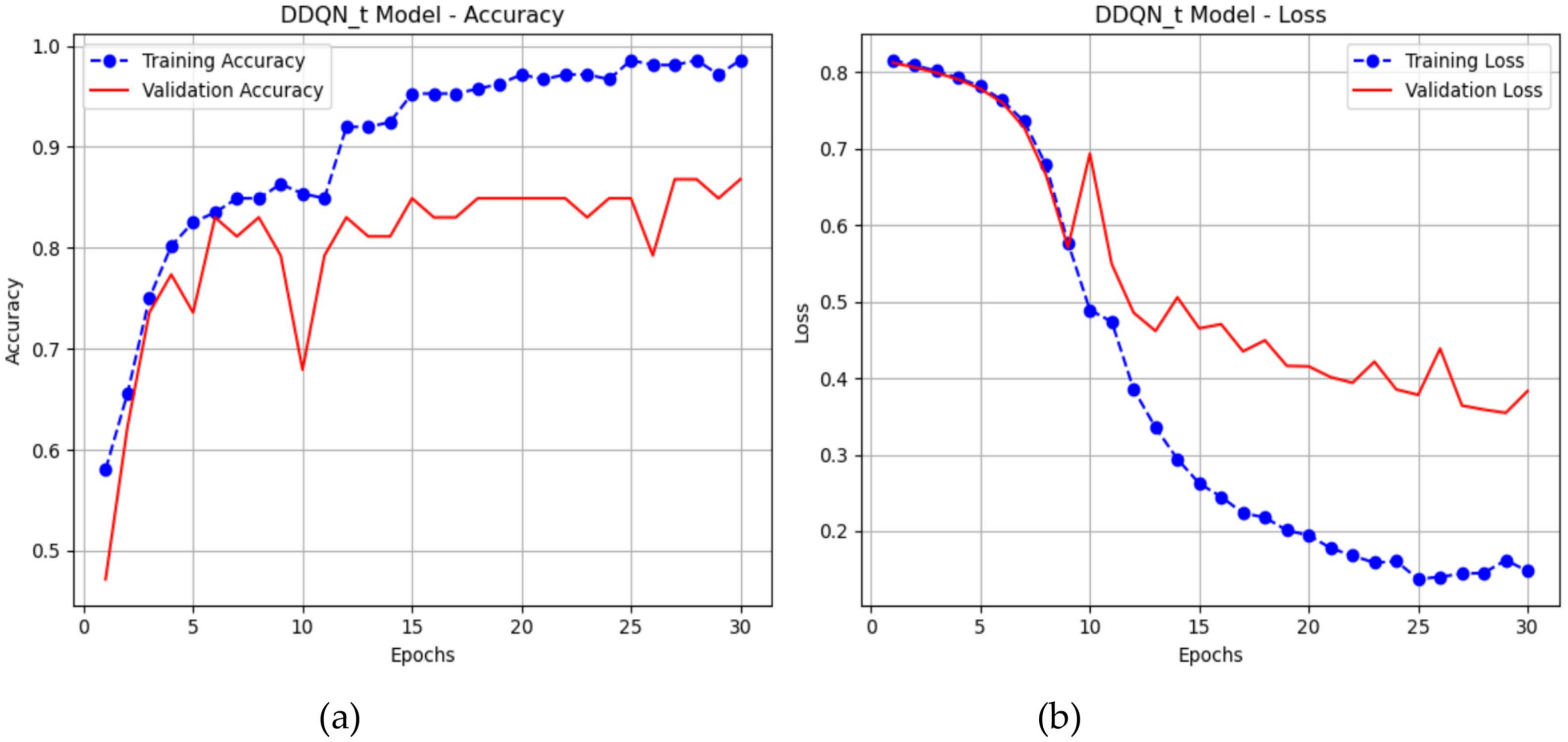
Performance of the *DDQN-inspired* model.

Graphs 16 illustrate the performance of a DDQN throughout 30 epochs. The accuracy plot (a) demonstrates that the training accuracy increases from around 58.02% to almost 98.58%, indicating the DDQN model successful learning from the training data over time. The validation accuracy of the DDQN is about 87, showing the best performance compared to different models like LSTM and CNN-LSTM. The plot (b) illustrates that the training loss decreases from about 0.8155 to around 0.1477, indicating a robust fit to the training data. The validation loss begins at 0.3831 with many fluctuations throughout (Figure 16).

**Figure 17:**
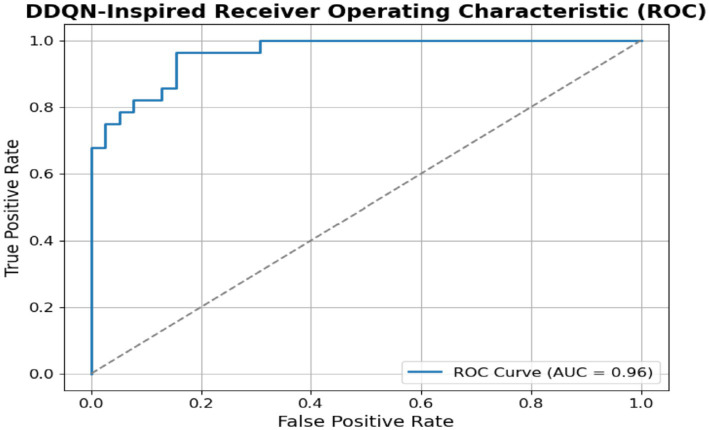
ROC *DDQN-inspired model.*

Figure 17 shows the ROC curve for the DDQN model; it shows a visual representation of its classification capability, with the curve toward the top-left corner, indicating strong predictive power. The AUC value of the DDQN model is 96%, demonstrating that the model can distinguish between the positive and negative classes.

## 4 Experiment and discussion results

Both the Jupyter deep learning framework and the Windows 10 operating system were utilized during the testing process. Experiments were conducted using a machine with 16 gigabytes of RAM and an Intel Core i7 central processing unit. The input dimensions of the experiment were a standard text dataset collected from the Twitter API related to ASD. The test was utilized in our database, while the remaining 20% was used as part of our validation set. The three DL models, namely LSTM, CNN-LSTM, and DDQN-Inspired, were proposed for detecting ASD from social media content.

### 4.1 Measuring the model’s performance

Sensitivity, specificity, accuracy, recall, and F1 scores are assessment measures used to determine how successfully the algorithms identify ASD. The related equations from 17 to 21:


(17) $$\text{Accuracy}=\frac{\mathrm{TP}+\mathrm{TN}}{\mathrm{TP}+\mathrm{FP}+\mathrm{FN}+\mathrm{TN}}\times 100\%$$

(18) $$\text{Sensitivity}=\frac{\mathrm{TP}}{\mathrm{TP}+\mathrm{FN}}\times 100\%$$

(19) $$\text{Precision}=\frac{\mathrm{TP}}{\mathrm{TP}+\mathrm{FP}}\times 100\%$$

(20) $$\text{specificity}=\frac{\mathrm{TN}}{\mathrm{TN}+\mathrm{FP}}\times 100\;$$

(21) $$F1-\text{score}=2*\frac{\text{precision}\times \text{Sensitivity}}{\text{precision}+\text{Sensitivity}}\times 100\;$$

### 4.2 Result of the LSTM model

The classification LSTM model, presented in Table 4, summarizes its performance in differentiating between ASD and Non-ASD patients, attaining an overall accuracy of 81%. The LSTM model demonstrates in ASD class a precision of 91%, indicating a high accuracy in identifying predicted ASD cases. The LSTM with recall metric scored 77% and an F1-score of 82% for detecting the ASD class. The LSTM model with Non-ASD class demonstrates a precision of 71%, a recall of 89%, and an F1-score of 79%, to identify Non-ASD cases. The macro average of the LSTM model for all metrics is (precision: 81%, recall: 82%, F1-score: 81%). LSTM model is recognized for its efficiency and scalability as a model for social media content.

**Table 4:** LSTM results.

Class name	Precision (%)	Recall (%)	F1 Score (%)	Support
ASD	91	74	82	39
Non-ASD	71	89	79	28
Accuracy		81		
Macro Avg	81	82	81	67

**Figure 18:**
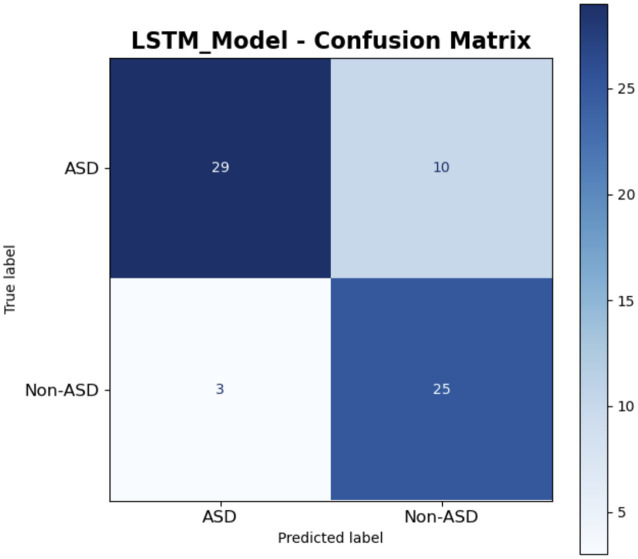
LSTM model.

The confusion matrix for the LSTM model is provided in Figure 18. It is presented in a clear manner. Among the confirmed ASD cases, 29 were accurately identified as ASD, whereas 10 were incorrectly classified as Non-ASD, indicating strong performance with minor errors. In the true non-ASD cases, 25 were correctly identified, while 3 were misclassified as ASD, suggesting a generally effective detection process. The deep blue and light shades produce a tranquil visual, illustrating the model’s balanced approach in classifying the 67 total instances, demonstrating notable strength in identifying Non-ASD cases, while exhibiting marginally lower accuracy for ASD. This matrix effectively illustrates the LSTM model’s systematic approach to managing sequential data, such as text or time-series inputs, in a clear and comprehensible manner.

### 4.3 Result of the CNN-LSTM model

Table 5 displays the CNN-LSTM model’s performance in distinguishing between ASD and non-ASD classes. The CNN-LSTM model attained an overall accuracy of 85% across the dataset. In the ASD label, a precision of 91% was achieved, a high percentage for predicting ASD cases that were accurately recognized. The recall indicates that the model identified 82% of all genuine ASD cases, resulting in an F1 score of 86%, better than the recall metric. The CNN-LSTM model attained 78% accuracy, 89% recall, and an 83% F1 score for the Non-ASD class. The macro average, representing the unweighted mean of precision, recall, and F1 score across both classes, was 85, 86, and 85%, respectively. The findings indicate that the CNN-LSTM model performs satisfactorily, exhibiting a marginally superior capacity to identify ASD cases relative to non-ASD cases accurately.

**Table 5:** Results of the CNN-LSTM model.

Class name	Precision (%)	Recall (%)	F1 Score (%)	Support
ASD	91	82	86	39
Non-ASD	78	89	83	29
Accuracy		85		
Macro Avg	85	86	85	67

**Figure 19:**
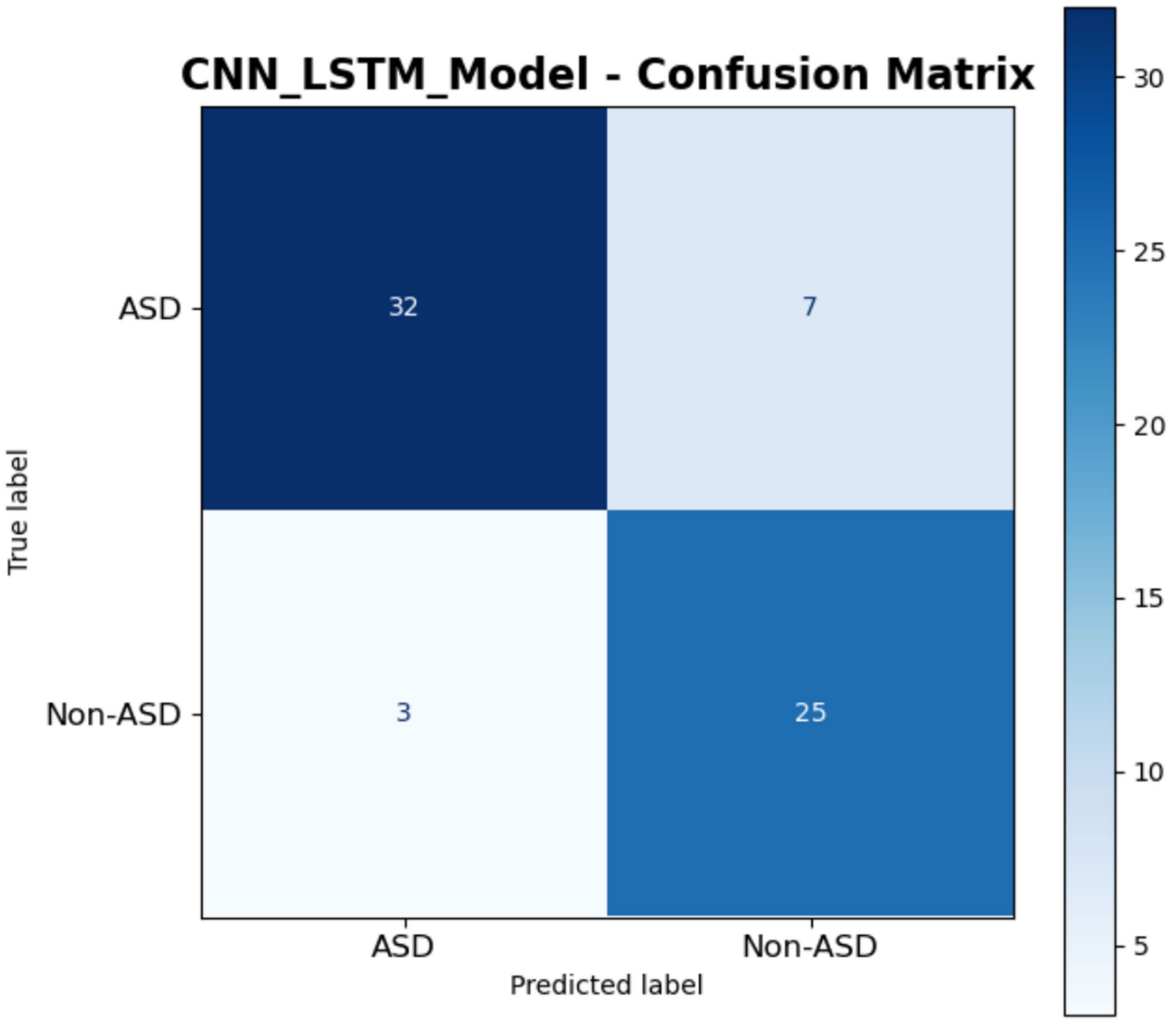
Results of CNN-LSTM model.

The confusion matrix of a CNN-LSTM model is presented in Figure 19, for classifying instances into ASD and Non-ASD. The matrix is structured with true labels on the vertical axis and predicted labels on the horizontal axis, providing a clear summary of the model’s classification outcomes. The matrix shows that out of the instances truly labeled as ASD, the model correctly predicted 32 as ASD TP while 7 were incorrectly classified as Non-ASD FN. For the instances truly labeled as Non-ASD, the model accurately identified 25 as Non-ASD TN but 3 were misclassified as ASD FP. This indicates that the model demonstrates a relatively strong ability to correctly identify ASD and Non-ASD cases, with higher accuracy for true positives (32 out of 39 ASD cases) and true negatives (25 out of 28 Non-ASD cases). Overall, the model exhibits promising performance with minimal misclassification errors.

### 4.4 Results of double deep Q-network

The findings of the DDQN model are shown in Table 6, achieving a high precision of 87% compared to the other models. This finding demonstrates the potential of the proposed DDQN approach for identifying ASD based on social media content. Ultimately, the proposed system was compared against the existing one using the same dataset. The proposed approach may assist physicians in detecting ASD and conducting symptomology research in a natural environment, attaining an overall accuracy of 87. The model for the ASD class shows a precision of 95%, a recall of 79%, and an F1-score of 87%, indicating robust efficacy in accurately identifying ASD patients. The Non-ASD class has a precision of 77%, a recall of 96%, and an F1-score of 86%, indicating somewhat reduced accuracy with robust recall. The macro average measures (precision 87%, recall 88%, F1-score 87%) indicate performance across both classes.

**Table 6:** Result of DDQN-inspired.

Class name	Precision (%)	Recall (%)	F1 Score (%)	Support
ASD	95	79	87	39
Non-ASD	77	96	86	28
Accuracy		87		67
Macro Avg	87	88	87	67

**Figure 20:**
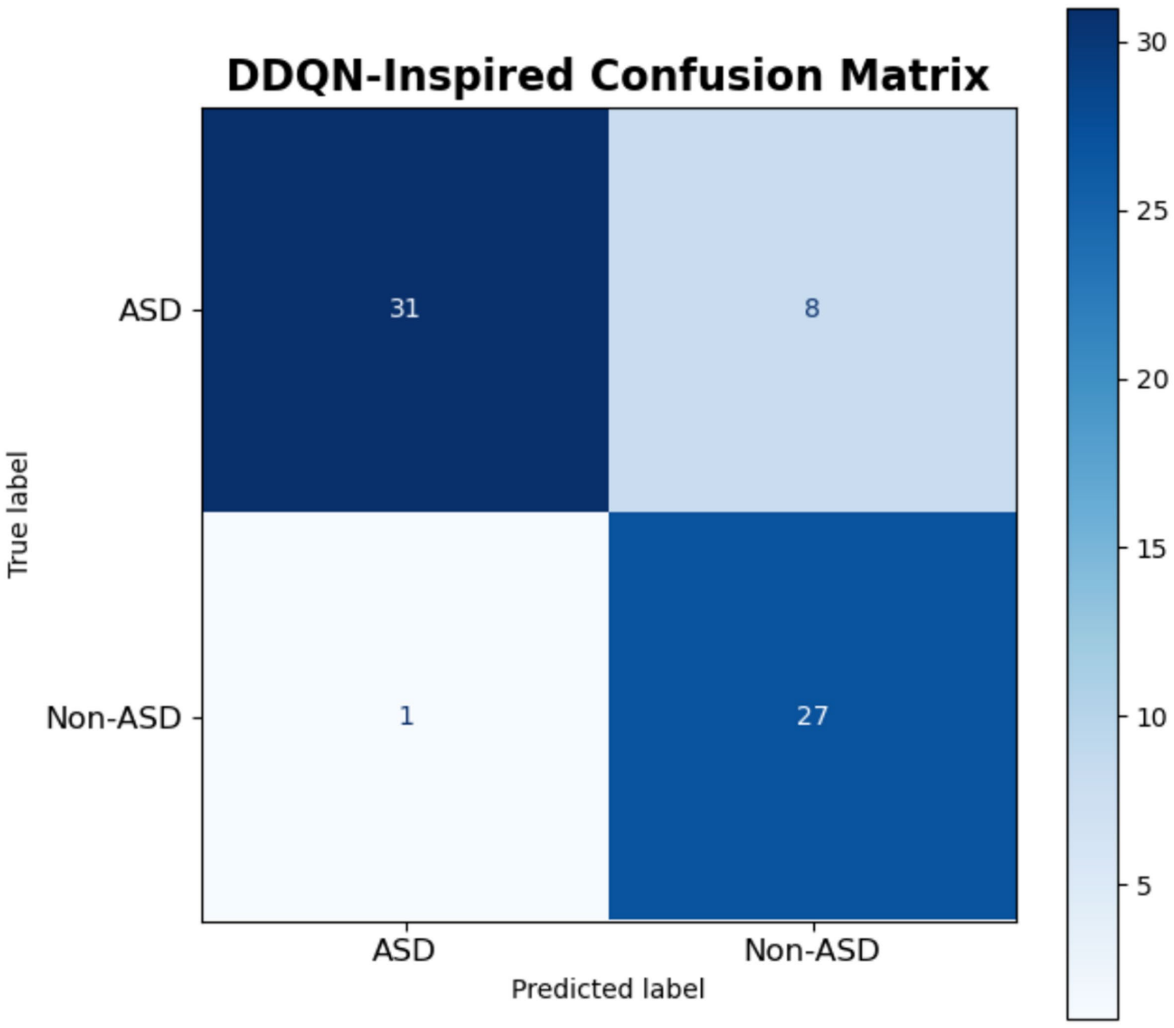
Result of DDQN-inspired model.

The confusion matrix of the DDQN model is shown in Figure 20 for the classification task between ASD and non-ASD cases. For correct classification of ASD cases, the model correctly classified 31 instances as ASD, represented by the top-left quadrant (TP). However, the DDQN model, misclassified 8 instances misclassifying true ASD cases as Non-ASD, shown in the top-right quadrant (FN). On the other hand, the DDQN showed the true Non-ASD cases, accurately identified 27 instances as Non-ASD, depicted in the bottom-right quadrant (TN). At the same time, 1 instance was incorrectly labeled as ASD, as shown in the bottom-left quadrant (FP). The confusion matrix of DDQN model highlights that it performs well overall, with a strong ability to correctly identify both ASD and Non-ASD cases, as evidenced by the high counts of TP (31) and TN (27).

In the digital era, people frequently write content on social media to express their feelings, opinions, beliefs, and activities. This makes social media one of the most significant sources of data generation, allowing you to explore its opportunities and challenges. Today, social media has become a mediator between people and the healthcare sector, enabling them to search for information about any specific disease and methods for diagnosing it.

**Figure 21:**
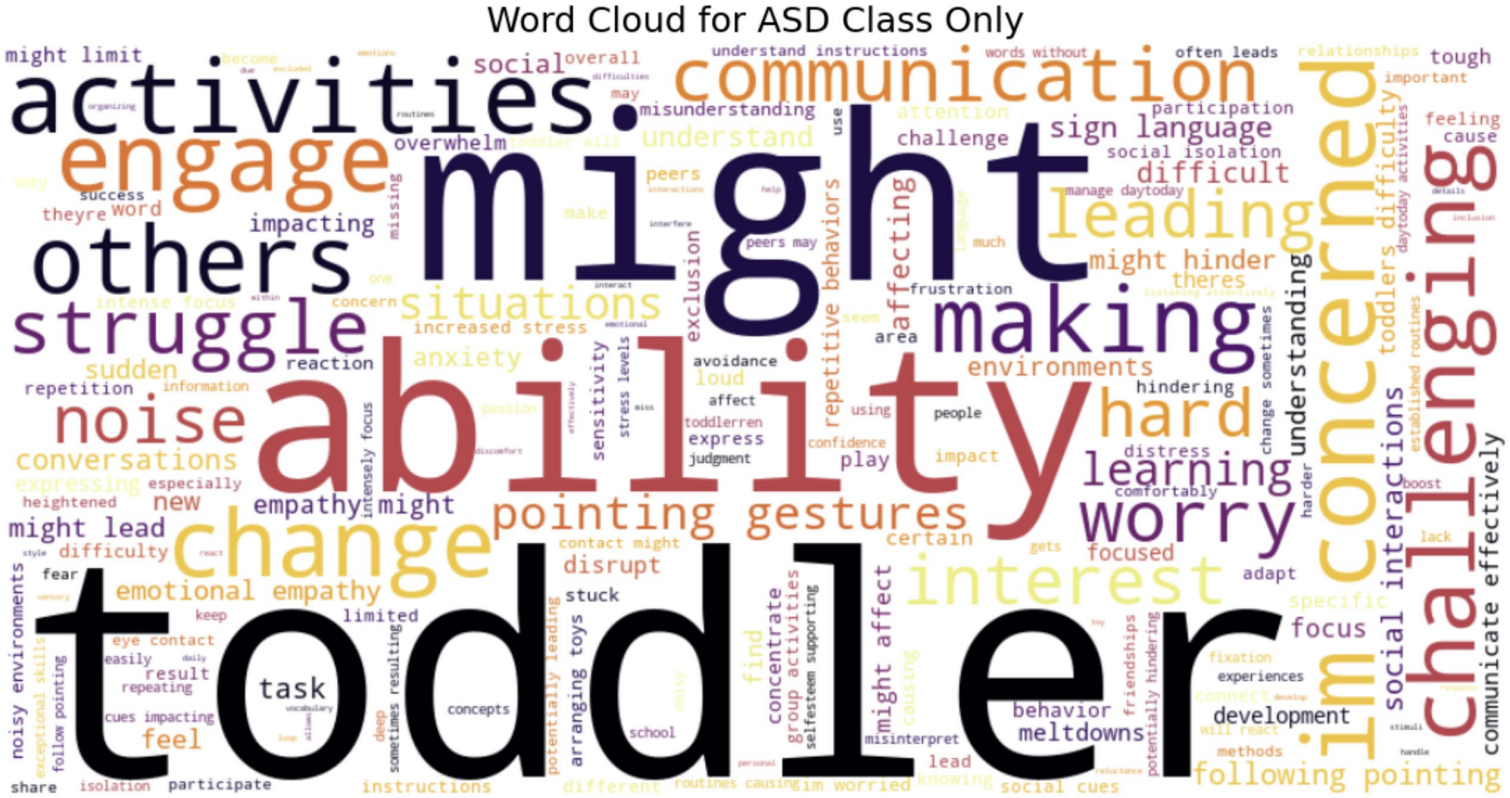
ASD word cloud.

Individuals within the mental health community use social media platforms such as Twitter to seek information, exchange experiences, and get assistance about ASD in an environment that is seen as more approachable and informal than conventional medical contexts. They often seek immediate, relevant information—whether to understand symptoms, identify coping mechanisms, or connect with others facing similar difficulties. Figure 21 illustrates that Word clouds are visual representations of text that highlight key terms and their frequency of use. We used WordCloud to compare ASD and Non-ASD texts for instances of word repetition.

**Figure 22:**
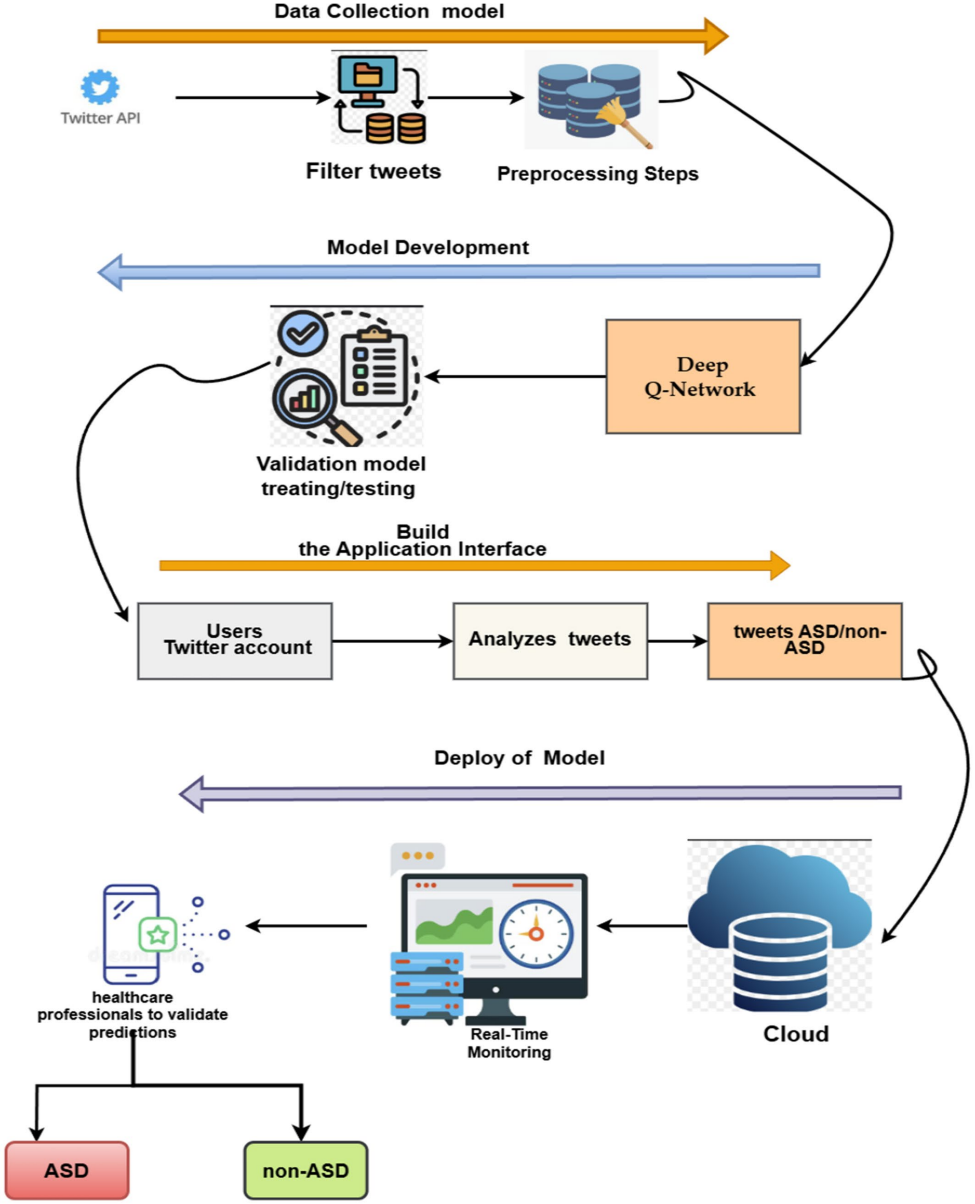
Deployment system-based text for detecting ASD.

The deployment model based on the Deep Q-Network (DQN) model for diagnosing ASD is shown in Figure 22.

Step 1: Data Collections, including cleaning, normalization, and tokenization.

Step 2: Model Development: The preprocessed data is used to train and validate a Deep Q-Network (DQN) model for classifying tweets as indicative of ASD or non-ASD patterns.

Step 3: Application Interface: An application interface is developed once the model has been trained. It integrates with users’ Twitter accounts and continuously analyzes their tweets.

Step 4: Deployment: The proposed system is deployed in the cloud for storing tweets, enabling real-time monitoring of incoming tweets. Predictions are flagged for review by healthcare professionals, who validate the model’s output before categorizing individuals as potentially having ASD or non-ASD.

This digital imprint may serve as an ancillary resource for mental health practitioners, providing insights into an individual’s emotional state and social behaviors in a natural environment, potentially facilitating early detection or corroborating a diagnosis. This method is a non-invasive means of data collection, particularly beneficial for individuals who lack rapid access to clinical assessments due to financial constraints, stigma, or resource scarcity. However, it should not replace professional diagnoses and must be conducted with ethical consideration to prevent misunderstanding. Table 7 shows the findings of the proposed framework on the Twitter dataset. It demonstrates that the suggested method outperforms the current systems in terms of accuracy, proving its efficacy and potential for performance improvements.

**Table 7:** Compared with the proposed ASD system.

References	Dataset	Model	ACC %
Rubio-Martín et al. (26)	Twitters dataset	BERT	84
Jaiswal and Washington (27)	Twitters dataset	ML	78

## 5 Conclusion

To assist people in identifying trends in their behavior, such as social challenges or sensory sensitivities, which may encourage them to pursue a formal diagnosis. The main objective of examining tweets for identifying ASD is its ability to provide behavioral and emotional indicators associated with the disorder. This research was used to analyze the textual analysis of tweets to detect the behaviors in self-identified autistic individuals relative to others. The suggested framework was evaluated using information from the social media platform “Twitter” collected from a public repository. Before examining the proposed system, several preprocessing steps must be implemented in the text. The ‘Text’ column is cleaned by converting it to lowercase, eliminating non-alphanumeric characters (excluding spaces) through regular expressions, normalizing whitespace to a single space, and removing any leading or trailing spaces. The ASD and Non-ASD labels are converted into a numerical format (0 or 1) with LabelEncoder to accommodate the binary classification requirement. Tokenization of the text data is performed using a tokenizer, restricting the vocabulary to 10,000 words, and then transforming the text into sequences of numbers. The sequences are padded to a standardized length of 200 tokens to maintain consistency for the proposed model input. The proposed data is ultimately divided into an 80% training and 20% testing ratio, and class weights are calculated to resolve any class imbalance. This preparation pipeline efficiently converts raw text data into a structured numerical representation appropriate for the proposed framework, while preserving academic integrity. The output of these preprocessing steps was processed using three DL models, such as Short-Term Memory (CNN-LSTM) and a Double Deep Q-network (DDQN). The results of these proposals were proven, revealing that the DDQN model achieved a high accuracy score of 87% with respect to the accuracy measure. The proposed framework, based on real textual data, can be helpful for real-time offering natural, behavioral, and emotional data that might indicate ASD-related characteristics. Finally, we have observed that social media (Twitter) postings include linguistic patterns, emotional expressions, and social interactions that can help official health officials detect ASD based on the thorough symptoms of ASD that are posted on the platform. This study utilized a conventional dataset sourced only from the Twitter network. We will emphasize the necessity of gathering datasets from many platforms to enhance the model’s generalizability in the future.

## Data Availability

The original contributions presented in the study are included in the article/supplementary material, further inquiries can be directed to the corresponding author/s. The dataset used in this study can be found at https://data.mendeley.com/datasets/87s2br3ptb/1.
